# Impact of hearing aid noise reduction algorithms on the speech-evoked auditory brainstem response

**DOI:** 10.1038/s41598-020-66970-2

**Published:** 2020-07-01

**Authors:** Hye Yoon Seol, Suyeon Park, Yoon Sang Ji, Sung Hwa Hong, Il Joon Moon

**Affiliations:** 1 Samsung Advanced Institute for Health Sciences & Technology, Sungkyunkwan University, Seoul, Korea; 2 Hearing Research Laboratory, Samsung Medical Center, Seoul, Korea; 3 Department of Otolaryngology-Head & Neck Surgery, Samsung Changwon Hospital, Sungkyunkwan University School of Medicine, Changwon, Korea; 4 Department of Otolaryngology-Head & Neck Surgery, Samsung Medical Center, Sungkyunkwan University School of Medicine, Seoul, Korea

**Keywords:** Sensory processing, Inner ear

## Abstract

The purposes of this study are to investigate the neural representation of a speech stimulus in the auditory system of individuals with normal hearing (NH) and those with hearing aids (HAs) and to explore the impact of noise reduction algorithms (NR) on auditory brainstem response to complex sounds (cABR). Twenty NH individuals and 28 HA users completed puretone audiometry, the Korean version of the Hearing in Noise Test (K-HINT), and cABR. In 0 and +5 dB signal-to-noise ratios (SNRs), the NH group was tested in /da/ only (quiet) and /da/ with white noise (WN) conditions while the HA group was tested in /da/ only, /da/ WN, /da/ WN NR ON, and /da/ WN NR OFF conditions. Significant differences were observed between /da/ only and /da/ WN conditions for F0 in both groups, but no SNR effect was observed for both groups. Findings of this study are consistent with previous literature that diminished cABR amplitudes indicate reduced representation of sounds in the auditory system. This is the first to examine the effect of a specific HA feature on cABR responses.

## Introduction

Hearing loss is a public health problem; the World Health Organization reports that 466 million people around the world experience disabling hearing loss^1^. For those who are diagnosed with sensorineural hearing loss, HAs that amplify acoustic signals are often recommended as a major rehabilitative option. HAs should be fitted appropriately based on each patient’s audiogram and characteristics, and a typical HA fitting appointment involves verification and validation. Patients are typically fitted with HAs with the aim of improving speech recognition in both quiet and noisy environments. Therefore, HAs contain features, such as NR, directional microphones, speech enhancement, or frequency lowering, to increase speech intelligibility.

Speech intelligibility is one’s ability to hear and understand speech signals. It is a natural, but complex process as both peripheral and central systems are involved^2–5^. Compared to speech recognition in noise, speech recognition in a quiet environment is less challenging for individuals with HAs^6,7^. When noise is present, speech signals get distorted, so it becomes challenging even for individuals with NH to understand speech^6,8^.

HAs have been shown to improve speech intelligibility in individuals with hearing loss. Hallgren, Larsby, Lyxell, and Arlinger (2005) compared the listening effort of HA users’ aided and unaided performance in quiet and noisy environments. Perceived effort was lower and word identification scores were higher with HA use^6^. However, there is a lack of evidence if various technical features employed in HAs are truly beneficial for speech understanding in noise. Brons, Houben, and Dreschler (2014) examined the effect of single-microphone NR on twenty individuals with sensorineural hearing loss. Although NR decreased annoyance caused by noise, it did not increase speech intelligibility; one of the randomly coded HA recordings in the study, NR2, was preferred the most by the participants, but showed the lowest scores for speech intelligibility^9^ Wu *et al*. (2019) investigated the effect of directional microphone and NR in basic and premium HAs using laboratory tests and self-reports. Laboratory tests showed that the features of the premium HAs aided in improving speech recognition and sound localization performance. Participants reported through self-reports that they felt more satisfied when they were able to use the features^10^.

Another issue for speech intelligibility is disparity between HA performance in clinical or laboratory environments and in the real-world, which has been noted in several studies^11–14^. While patients complete clinical assessments in a soundproof booth without visual cues, the real world is not as controlled as the sound booth in clinic. The real world is much noisier and visual cues, such as lip movements, are provided simultaneously to potentially improve communication. Due to this mismatch, an accurate evaluation of the effectiveness of HA features is challenging and the efforts to create a laboratory environment that better reflects real world listening situations are ongoing^15^. For NR, some studies examined its effectiveness objectively using pupillometry^16–18^, but research into the effect of NR using auditory brainstem response to complex sounds (cABR) has not been conducted yet.

Hearing could also be tested using electroencephalography (EEG). In general, the auditory brainstem response (ABR), a type of EEG signal generated by the auditory nervous system in response to an acoustic stimulus, is utilized to assess the integrity of the auditory pathway, more specifically, from the cochlea to the lower part of the brainstem^19^. ABR is commonly used in individuals who are not able to provide reliable feedback (i.e. individuals with disabilities and infants) to measure their hearing sensitivity and for site-of-lesion testing and intraoperative monitoring^20,21^. Click and tone-burst are typically used to elicit ABR responses, but these stimuli do not reflect how complex speech stimuli are processed in the auditory system.

In recent years, ABR using complex sounds, cABR, has been gaining more traction as it is an objective way to assess neural encoding with close to real-world stimuli compared to traditional stimuli which are clicks and tone bursts^22^. cABR contains responses to transient and sustained regions of the stimulus. The sustained response is phase-locked to the periodic information of the stimulus below 1000 Hz and thus, it is often called a frequency following response (FFR)^23,24^. Transient responses reflect higher formant information^25^. Similar to ABR, cABR is passive meaning that patients do not need to raise their hand or say “yes” to indicate that they heard and understood speech. Therefore, cABR could also be performed on difficult-to-test populations for diagnostic testing and outcome assessments. Many studies have mentioned the feasibility of cABR as a clinical tool^21,26–30^.

Along with behavioral assessments, evaluating higher-level processes through cABR could provide insight to any underlying mechanisms of the neural representation of speech signals in the central auditory system. In Nada, Kolkaila, Gabr, and El-Mahallawi (2016), 60 participants were divided into control and study groups and completed auditory brainstem testing with click and speech stimuli. The control group consisted of participants with NH and the study group consisted of participants with bilateral mild to moderate sensorineural hearing loss. The results showed no effect of hearing loss on cABR amplitudes. However, hearing loss led to delayed latencies indicating disturbed neural synchronization^31^. In terms of aided cABR, Easwar, Purcell, Aiken, Parsa, and Scollie (2015) reported that testing parameters, such as stimulus level, bandwidth, and use of hearing technology, affect cABR responses. With amplification, the number of FFRs detected significantly increased^32^. Jenkins, Fodor, Presacco, and Anderson (2018) observed changes in FFRs with amplification. Between unaided and aided conditions, only 65 dB SPL presentation level, among three presentation levels (65 dB SPL in quiet, 80 dB SPL in quiet, and 80 dB SPL with 70 dB SPL of 6-talker babble), showed increased phase-locking and amplitudes and decreased latencies^33^. Lastly, BinKhamis, Elia Forte, Reichenbach, O’Driscoll, and Kluk (2019) reported larger peak and F0 coding amplitudes and earlier peak latencies with aided speech-ABRs^34^. There are also studies that explored the relationship between speech in noise performance and cABR as speech understanding in noise is one of the most common complaints from HA users^35–37^. Comparison of cABR responses was performed in Anderson, Parbery-Clark, Yi, and Kraus (2011) with 28 adults with NH. The participants completed the Hearing in Noise Test (HINT) and were divided into top and bottom groups based on their HINT performance. Comparing the top and bottom groups’ cABR responses, the bottom group’s responses showed decreased neural representation of the stimulus and their responses were affected more greatly by noise^38^.

Expanding on prior studies, the purposes of this study are to investigate the neural representation of a speech stimulus in NH and HA groups and explore the impact of HA NR algorithms on cABR. We hypothesized that hearing loss and background noise will influence cABR responses in a way that they will reduce the amplitudes of the responses. We hypothesized that when the HA NR function is activated, better cABR responses would be observed as participants would be able to hear the stimulus better in noise. Ultimately, findings about whether or not the HA NR algorithms affect cABR responses in HA users will provide information about how effective the feature is to help individuals to process the acoustic inputs to perform complex listening tasks.

## Results

**Figure 1:**
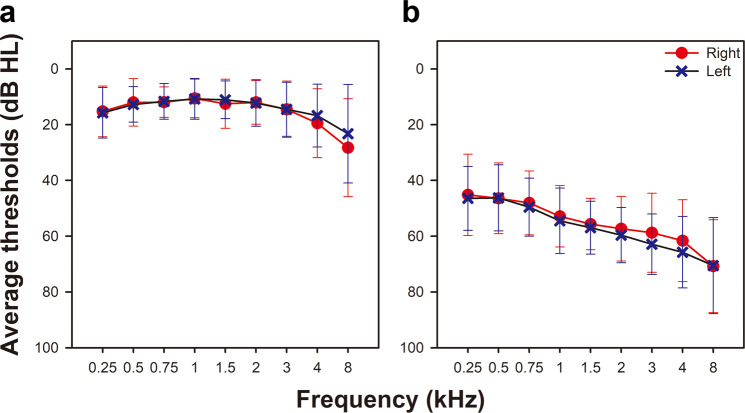
Average pure tone thresholds for NH listeners (left) and HA users (right) with ±1 SD. Red circles indicate the mean air conduction thresholds for the right ear while blue crosses represent the mean air conduction thresholds for the left ear across frequencies.

The age range of the participants was from 19 to 81 years old. Mean ages of the NH and HA group were 53.45 years (SD = 16) and 56.35 years (SD = 17.2), respectively. Pure tone averages of the NH group were 13.5 dB in the right ear and 13.1 dB in the left ear. Participants in the HA group had moderate to moderately severe sensorineural hearing loss bilaterally. Pure tone averages of the HA group were 54.5 dB in the right ear and 56.5 dB in the left ear. Average pure tone thresholds for both groups are shown in Fig. 1.

### K-HINT

**Figure 2:**
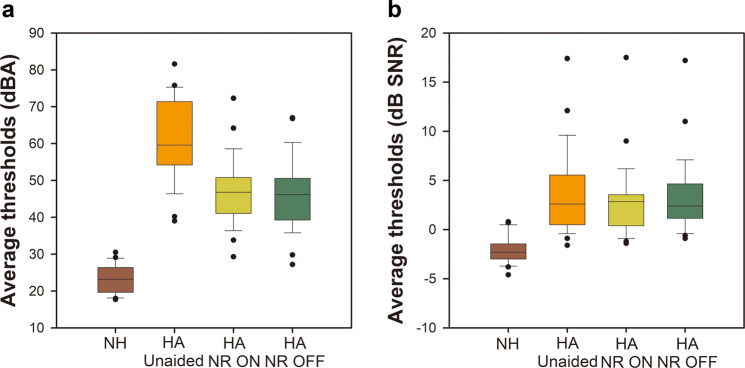
Mean K-HINT performance in quiet (left) and noise from the front (right) for both groups with ±1 SD. The horizontal lines within the shaded bars represent the median values. Shaded bars represent interquartile ranges and the highest and lowest points are displayed by the error whiskers. Filled circles beyond these points are outliers.

K-HINT was performed for all individuals who participated in this study. Figure 2 illustrates participants' K-HINT performance in quiet and noise from the front conditions. In the quiet condition, NH listeners were able to repeat K-HINT sentences at a lower reception threshold level than HA users. Statistical analysis using the Wilcoxon signed-rank test revealed that activation of NR function did not have any impact on HA users’ K-HINT performance (p-value = 0.2512, Z statistics=1.13, Degrees of freedom(DF) = 27). In the noise condition, both NH listeners and HA users experienced difficulty understanding sentences, but NH listeners still had a lower reception threshold for sentences. With hearing loss, HA users’ reception thresholds were higher than those of NH listeners meaning that the presentation level of the sentences had to be higher in order for the HA users to correctly repeat back the sentences. NR also did not have any impact on K-HINT performance; the reception thresholds for sentences were similar regardless of the activation of the feature. Statistical analysis using the Wilcoxon signed-rank test also revealed no significant difference between the aided NR ON and aided NR OFF conditions for HA users in noise (p-value = 0.3647, Z statistics = −0.91, DF = 27).

### The impact of noise on cABR responses in NH listeners

**Figure 3:**
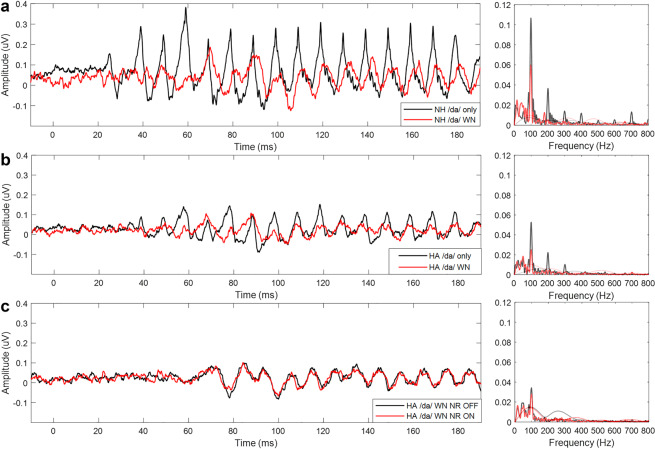
(**a**) NH listeners’ cABR responses and FFT analysis in the /da/ only (black) and /da/ WN (red) conditions. (**b**) HA users’ cABR responses and FFT analysis in the /da/ only (black) and /da/ WN (red) conditions. (**c**) HA users’ cABR responses and FFT analysis in the /da/ WN NR OFF (black) and /da/ WN NR ON (red) conditions. For all FFT analysis, noise floor for the /da/ only and /da/ WN NR OFF conditions are displayed in grey and /da/ WN and /da/ WN NR ON conditions are displayed in pink.

Figure 3 displays grand average waveforms of the cABR responses of all participants. Fourier analysis which represents 60 to 170 ms portion of the stimulus in frequency domain is also shown in the figure. The negative impact of noise is well shown in NH listeners’ cABR responses in the /da/ only and /da/ WN conditions (Fig. 3a)—when noise was present, their cABR amplitudes were reduced. The Wilcoxon signed-rank test indicated that there was a statistically significant difference for F0 (p-value = <0.0001, Z statistics=3.66, DF = 19) in the /da/ only and /da/ WN conditions.

### The impact of SNR on cABR responses in NH listeners

In regard to SNR, the Mann-Whitney U test revealed no significant difference for the NH group for F0 in the /da/ WN condition (p-value = 0.579, Z statistics = −0.55, DF = 18).

### The impact of noise on cABR responses in HA group

HA users’ cABR responses were also affected by noise in /da/ only and /da/ WN conditions (Fig. 3b). Statistical analysis of the HA users’ cABR responses was completed with the Wilcoxon signed-rank test. In order to correct for multiple comparisons, the Bonferroni correction was applied for all p values (p < 0.0083). There was a significant difference for F0 between the two conditions (p-value <0.0001, Z statistics=3.69, DF = 27).

### The impact of SNR on cABR responses in HA group

The Mann-Whitney U test revealed no significant difference for F0 in the /da/ WN condition (p-value=0.945, Z statistics=0.07, DF = 26).

### Comparison of cABR responses between NH and HA groups

Both groups’ cABR responses were compared using the Mann-Whitney U test. The results showed significant differences for F0 in the /da/ only (p-value=0.0058, Z statistics=2.76, DF = 46) and /da/ WN conditions (p-value=0.0072, Z statistics=2.69, DF = 46).

### The impact of HA NR function in HA group

HA users’ cABR responses with the NR function turned on and off were examined (Fig. 3c). The Wilcoxon signed-rank test revealed no statistical significance between the /da/ WN NR OFF and /da/ WN NR ON conditions for F0 (p-value = 0.2463, Z statistics = 1.20, DF = 27).

### Correlation between cABR fundamental frequency amplitude of F0 amplitude and K-HINT performance in NH listeners

**Figure 4:**
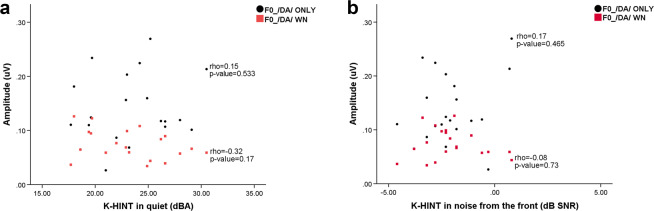
(**a**) Correlation between K-HINT performance in quiet and F0 amplitudes in /da/ only (black circle) and /da/ WN conditions (red square) (**b**) Correlation between K-HINT performance in noise from the front and F0 amplitudes in /da/ only (black circle) and /da/ WN conditions (red square).

The Spearman correlation test was performed to examine the correlation between the NH listeners’ cABR fundamental frequency amplitude of F0 amplitude and K-HINT performance (Fig. 4). The results revealed that there was no significant correlation between the cABR fundamental frequency amplitude of F0 amplitude in /da/ only and K-HINT performance in quiet (p-value=0.533, Rho=0.15). The correlation between the fundamental frequency amplitude of F0 amplitude in /da/ only and K-HINT performance in noise was also not significant (p-value=0.465 Rho=0.17). No significant correlations were found between the cABR fundamental frequency amplitude of F0 amplitude in /da/ WN and K-HINT performance in quiet (p-value=0.17, Rho=−0.32) or for the cABR fundamental frequency amplitude of F0 amplitude in /da/ WN and K-HINT performance in noise (p-value=0.73, Rho=−0.08).

### Correlation between cABR fundamental frequency amplitude of F0 amplitude and K-HINT performance in HA group

**Figure 5:**
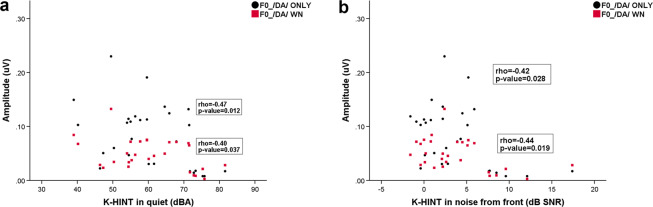
(**a**) Correlation between K-HINT performance in quiet and F0 amplitudes in /da/ only (black circle) and /da/ WN conditions (red square) (**b**) Correlation between K-HINT performance in noise from the front and F0 amplitudes in /da/ only (black circle) and /da/ WN conditions (red square). All values were statistically significant (bold).

Figure 5 displays the correlation between the cABR fundamental frequency amplitude of F0 amplitude and K-HINT performance in the HA group. The Spearman correlation test showed significant correlations between the cABR fundamental frequency amplitude of F0 amplitude in /da/ only and K-HINT performance in quiet (p-value=0.012, Rho=−0.47) and in noise (p-value=0.028, Rho=−0.42). Significant correlations were also identified between the cABR fundamental frequency amplitude of F0 amplitude in /da/ WN and K-HINT performance in quiet (p-value=0.037, Rho=−0.40) and in noise (p-value=0.019, Rho=−0.44).

### Correlation between cABR fundamental frequency amplitude of F0 amplitude and K-HINT performance in HA group with the NR function on and off

**Figure 6:**
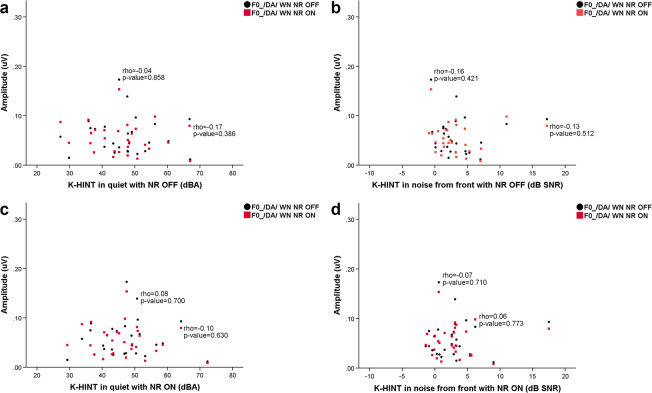
(**a**) Correlation between K-HINT performance in quiet with NR OFF and F0 amplitudes in /da/ WN NR OFF (black circle) and /da/ WN NR ON conditions (red square). (**b**) Correlation between K-HINT performance in noise from the front with NR OFF and F0 amplitudes in /da/ WN NR OFF (black circle) and /da/ WN NR ON conditions (red square). (**c**) Correlation between K-HINT performance in quiet with NR ON and F0 amplitudes in /da/ WN NR OFF (black circle) and /da/ WN ON conditions (red square). (**d**) Correlation between K-HINT performance in noise from the front with NR ON and F0 amplitudes in /da/ WN NR OFF (black circle) and /da/ WN NR ON conditions (red square).

Figure 6 shows correlations between the cABR fundamental frequency amplitude of F0 amplitude and K-HINT performance in the HA group with the NR function on and off. The results are also summarized in Table 1. In the /da/ WN NR OFF condition, the Spearman correlation test showed no significant correlations between the cABR fundamental frequency amplitude of F0 amplitude and K-HINT performance with NR ON in quiet (p-value=0.700, Rho=0.08) or for the cABR fundamental frequency amplitude of F0 amplitude and K-HINT performance with NR ON in noise (p-value=0.710, Rho=−0.07). No significant correlations were also found between the cABR fundamental frequency amplitude of F0 amplitude in /da/ WN NR OFF and K-HINT performance with NR OFF in quiet (p-value=0.858, Rho=−0.04) or for noise (p-value=0.421, Rho=−0.16). In the /da/ WN NR ON condition, no significant correlations between the cABR fundamental frequency amplitude of F0 amplitude and K-HINT performance in quiet with NR ON (p-value=0.630, Rho=−0.10) or for the cABR fundamental frequency amplitude of F0 amplitude and K-HINT performance in noise with NR ON (p-value=0.773, Rho=0.06). Lastly, the results showed no significant correlations between the cABR fundamental frequency amplitude of F0 amplitude in /da/ WN NR ON and K-HINT performance in quiet with NR OFF (p-value=0.386, Rho=−0.17) or for the cABR fundamental frequency amplitude of F0 amplitude in /da/ WN NR ON and K-HINT performance with NR OFF in noise (p-value=0.512, Rho=−0.13).

**Table 1:** Correlational analysis between K-HINT and fundamental frequency amplitude of F0 amplitude in the HA group with the NR feature on and off.

K-HINT	F0 amplitude	Rho	p-value
NR OFF in quiet	/da/ WN NR OFF	−0.04	0.858
	/da/ WN NR ON	−0.17	0.386
NR OFF in noise from the front	/da/ WN NR OFF	−0.16	0.421
	/da/ WN NR ON	−0.13	0.512
NR ON in quiet	/da/ WN NR OFF	0.08	0.700
	/da/ WN NR ON	−0.10	0.630
NR ON in noise from the front	/da/ WN NR OFF	−0.07	0.710
	/da/ WN NR ON	0.06	0.773

## Discussion

Ever since 1996, when digital signal processing was introduced for the first time^39^, hearing technology has advanced over the years to aid HA users in understanding speech in noise better than they were able to before. Examples include directional microphones, NR, ear-to-ear communication, hearing aid accessories, and so on^40,41^. However, even with the help of HAs, due to various reasons (i.e. impaired peripheral auditory system), acoustic signals are not fully and clearly represented in their central auditory system. This makes communication extra challenging as communication requires fast processing of auditory and visual cues. Oftentimes, individuals with hearing loss expect that HAs will restore their hearing and they will be able to hear and understand speech clearly again. When they realize that they actually need an adjustment period for at least a few months, they feel frustrated and sometimes return the HAs because they still “cannot hear well” with HAs. Besides, when assessing HA benefits, current behavioral test results, such as aided thresholds or aided speech performance, as well as real-ear measurements do not truly reflect HA users’ performance in the real world. For aided soundfield thresholds, the stimuli are often narrow-band noise which is not the sound we hear in our every communication environment. In terms of speech in noise performance, test results obtained in a soundproof booth do not necessarily show how sounds are represented in the central auditory system.

The current study investigated the impact of noise and HA NR algorithms on cABR responses in NH individuals and HA users. Our results revealed that the cABR amplitudes are reduced with background noise for both individuals with NH and hearing loss illustrating that the acoustic signals are less represented in their auditory system. No SNR effect was observed on both groups’ cABR responses which is consistent with findings from Lagace, Koravand, Thompson, and Lteif (2016) study^42^. Significant correlations were found between the fundamental frequency of F0 amplitude and K-HINT performance in quiet and noise in the HA group in the /da/ only and /da/ WN conditions.

Although these findings are consistent with previous literature to some extent that reduced cABR amplitudes indicate poor representation of sounds at the level of the brainstem^32,33,43–47^, there are mixed findings in terms of the effects of noise, SNRs, and amplification on the cABR responses, especially for those with hearing loss^33,34^. For example, Jenkins, Fodor, Presacco, and Anderson (2018) used a /ga/ syllable to examine adults’ cortical and subcortical responses in quiet and noise with and without HAs. For the subcortical responses, use of HAs led to increased amplitude and decreased latency when the stimulus was presented at 65 dB SPL^33^. The authors found no noise effects on subcortical response. However, significant effects of noise were found for cortical responses with increased N1 and P2 latencies and decreased P2 amplitude. Although these findings are somewhat similar to the findings of our study, it is important to note that a different SNR value (+10 dB SNR vs. 0 and +5 dB SNR), type of noise (6-talker babble vs. white noise), HA, and type of stimulus (/ga/ vs. /da/) were used. Regarding K-HINT performance, significant correlations were observed between the cABR fundamental frequency of F0 amplitude and K-HINT performance in quiet and noise in the /da/ only and /da/ WN conditions in the HA group. However, activation of NR did not have any influence on speech performance of participants who wore HAs. Again, the results are consistent with previous literature to some extent^11,13,25,38^, but there are studies showing no relationships between the cABR responses and speech performance. Further studies with multiple noise conditions instead of only one (noise from the front) would be helpful to further explore the effects of NR.

There are studies that have examined the relationships between behavioral measures in individuals with NH and hearing loss^48,49^. Mai, Tuomainen, and Howell (2018) investigated the relationship between speech in noise performance and cortical sensitivity (Theta-band phase-locking values) towards temporal acoustic cues using scalp EEG in steady-state speech-shaped noise and 16-speaker babble noise conditions. Results revealed that in the steady-state speech-shaped noise condition, better speech performance as well as higher theta-band phase-locking values were observed^48^. The effect of aging on speech performance, FFR, and magnetoencephalography (MEG) responses was examined in Presacco, Simon, and Anderson (2019) in quiet and noise conditions. In this study, there were no significant differences between older adults with NH and older adults with hearing loss for FFR and MEG, suggesting that temporal processing deficits could play a role in communication difficulties along with hearing loss^49^. Currently, literature investigating the effect of noise and use of amplification on HA users’ cABR responses is sparse and therefore, additional studies are necessary and careful interpretations should be made when interpreting the findings.

Although we hypothesized that the NR function would have a positive impact on the cABR responses for both F0, no NR effect was found for F0. It is difficult to pinpoint exactly why there was no NR effect, but some possible explanations include duration of HA use, HA types, HA fitting formula, lack of real-ear measurement, and noise types. Some studies claim that cABR responses are subjective to experience, such as auditory training and musical experience. In Kraus and Chandrasekaren (2010), musicians showed enhanced cABR responses towards music, working memory, and speech in noise performance when compared to those of non-musicians. Findings indicate that experiences that enrich our sensory modalities can lead to neuroplasticity and strengthen cABR responses^50,51^. The benefit of NR may depend on the type of noise used for the testing. For example, Wong, Chen, Wang, and Kuehnel (2018) examined the impact of NR on speech reception thresholds with steady-state noise and demonstrated a significant improvement in the thresholds with the activation of NR^52^. Regarding HA types, some participants originally wore completely-in-the-canal and behind-the-ear HAs and they were all fitted with RIC HAs for this study. Similarly, some participants needed to get used to the new fitting formula as we fitted all participants with RIC HAs with NAL-NL 2 formula. Thus, it is difficult to generalize the findings to all clinical populations^33^ and further studies with more variety in participant characteristics are necessary.

We, along with many other studies, believe that cABR has the potential to become a clinical tool. With speech as a stimulus, cABR demonstrates how the real-world stimuli are encoded at the level of the brainstem and could be used along with other behavioral test results for counselling. HA users will have more realistic expectations, such as acknowledging that speech understanding in background noise will still be challenging with HAs and are encouraged to actively use communication strategies. In addition, clinicians may be able to incorporate the cABR results into HA fitting and adjustment appointments to accomplish better HA optimization, resulting into shorter HA adaption period and increased HA satisfaction. In the long run, it might decrease the financial burden for some patients who have to travel far to see their audiologists. Increased HA satisfaction will lead to less HA return rate, loyalty to healthcare professionals, and increased willingness to recommend HAs to those in need. Adult HA users will ultimately have better access to interpersonal stimulation and engagement along with decreased listening effort for communication. They will be able to participate in many social or occupational opportunities that they once were restricted to participate due to hearing impairment.

However, it is important to note that significant amount of work is needed to verify the objectivity and reliability of cABR. As mentioned in BinKhamis *et al*. (2018) and Novis and Bell (2018) studies, normative data in quiet and noise in a variety of clinical populations as well as reliability of cABR responses should be further collected and investigated^21,30^. Novis and Bell (2018) compared the quality of cABR responses with click ABR and found that cABR responses had lower quality of responses^30^. The amplitudes of the cABR responses were significantly lower than the click ABR responses. The consistency of cABR peaks was also not found in all participants while wave V of click ABR was identified in all participants. However, in Bidelman, Pousson, Dugas, and Fehrenbach (2018) which used a 100-ms vowel /a/, FFR amplitudes were found to be stable^53^.

No quantification of data quality in individual participants was measured in this study either. BinKhamis *et al.* (2019) reported the possibility of using a shorter stimulus (40-msec) reliably by examining the impact of stimulus duration, noise, three consonant-vowels that are frequently used in cABR studies^47^. Quality of individual data needs to be investigated in subsequent studies as individual data will be collected in clinical settings. Test time is another potential limitation for cABR. Compared to speech testing which is also noninvasive, electrophysiological testing generally takes longer time because electrodes need to be applied and several thousand sweeps need to be taken to obtain reliable morphology of the waveforms. Therefore, although cABR does have the potential to be implanted in clinical settings, more studies investigating these issues are needed.

To the best of our knowledge, our study is the first to examine the effect of a specific HA feature on individuals’ cABR fundamental amplitudes. Although we only investigated the impact of NR function on individuals’ brainstem neural encoding, with the growing interest in cABR’s potential to be a valid clinical tool, we hope we paved the way to further explore the effect of various HA features, such as directional microphone and frequency lowering, on cABR responses. We can not only assess the impact of HA use, but also the impact of HA features on individuals’ cABR responses objectively. We believe that cABR would be beneficial for both healthcare professionals and adult HA users to objectively assess HA benefits. Once enough data has been gathered and norms are established, cABR could be performed on difficult-to-test populations who cannot provide reliable subjective feedback or who might not be cooperative during sound booth testing.

Further studies with larger sample sizes are necessary. Variety in participant characteristics, such as different configurations and severity of hearing loss, duration of hearing loss, types of HAs, cognitive function, and past auditory or musical training experience, would be beneficial. Other components of cABR (ex. latency) should be analyzed as well. Bellier *et al*. (2015) compared two methods (delivering signals directly to HAs vs. insert earphones) of obtaining cABR responses and reported that better cABR responses were obtained without artifacts when the signals were delivered directly to the HAs^54^. We could try streaming the stimulus to the HAs for subsequent studies. Lastly, additional studies could explore the underlying mechanisms of NR on cABR responses in HA users and the impact of other HA features, such as frequency lowering and directional microphones. The quality and reliability of individual cABR recordings is also important to assess before the methods can be used for clinical applications.

## Methods

### Participants

A total of 48 individuals participated in the study: 28 individuals with bilateral sensorineural hearing loss who wear HAs and 20 participants with NH. The participants with NH had average audiometric pure-tone thresholds below 25 dB HL at 500, 1000, 2000, and 4000 Hz with no asymmetry in hearing thresholds exceeding 10 dB at any of the frequencies tested. The participants with HAs had average audiometric pure-tone thresholds above 41 dB HL at 500, 1000, 2000, and 4000 Hz with no asymmetry in hearing thresholds exceeding 10 dB at any of the frequencies tested. Only those with bilateral sensorineural hearing loss were recruited for this study. All participants were native speakers of Korean. Exclusion criteria included individuals who were unable to communicate and understand TV at a distance of 1 m and cases with neurological and mental disorders. All participants traveled to Samsung Medical Center for testing and received payment per diem. All experimental procedures were approved by the regulations set by Samsung Medical Center’s Institutional Review Board and were carried out in accordance with approved guidelines. All participants signed an informed consent document prior to testing.

### Conventional Puretone Audiometry

Conventional pure-tone audiometry was performed in a sound booth using one of the following audiometers with TDH-39 headphones: Orbiter 922, GSI 61, or Aurical. Pure-tone audiometry was performed using the 2005 American Speech-Language-Hearing Association guidelines^55^.

### Hearing aid fitting

A pair of GN Resound LINX 2 RIC hearing aids were programmed with closed domes and NAL-NL 2 formula to fit all participants in our study prior to testing. Hearing aid fitting was performed by licensed research audiologists. Real-ear measurements were not performed in this study. Electroacoustic measurement was also completed using a hearing aid test box to verify that the NR feature was working properly. When the NR feature was activated, the signal was reduced by 6 dB. Average duration of HA use ranged from 0.5 months to 144 months.

### K-HINT

K-HINT was developed by Sung Kyun Moon and his colleagues at Ajou University and House Ear Institute and is widely used in clinical settings in Korea to assess one’s speech understanding ability in quiet and noisy environments^56^. K-HINT consists of twelve lists and each list contains twenty sentences. In our study, all stimulus presentation and data collection were completed through the use of HINT pro 7.2 (Natus, USA). Sentences were presented binaurally through a speaker in front of participants (distance: 1 m) in a semi-anechoic chamber. Participants were asked to face the speaker and remain motionless during each stimulus presentation for K-HINT. Participant placement was set up prior to each trial and monitored with a camera placed in the semi-anechoic chamber. In the quiet condition, only the K-HINT sentences were presented through the speaker located in front of participants. The starting level was 60 dBA. In the front noise condition, the sentences as well as the noise were presented from the same speaker located in front of the participants. The starting SNR was 0 dB with 65 dBA speech and 65 dBA noise levels. If the participant correctly repeated the sentence, the speech level was decreased by 4 dB. If the participant incorrectly repeated the sentence, the speech level was increased by 2 dB. The noise level was kept constant at 65 dBA. The NH group completed K-HINT in quiet and front noise conditions while the HA group completed the test in six conditions: unaided quiet, unaided noise from the front, aided quiet NR ON, aided quiet NR OFF, aided noise from the front NR ON, aided noise from the front NR OFF. Different lists were presented for each participant as well as each condition. The testing order was the same for all participants; the participants started with the quiet condition first and then the noise condition.

### cABR

cABR responses were obtained through Neuroscan SynAmps2 and STIM2 (Compumedics, Inc., USA). In terms of cABR recording parameters, we took recommendations provided by Skoe and Kraus (2010). Four electrodes were placed after carefully cleaning the placement sites. The ground electrode was placed on the forehead. The active electrode was placed on Cz with the reference electrodes on the earlobes. A 170-ms /da/ that was synthesized in a Klatt-based synthesizer with the fundamental frequency of 100 Hz was provided by Nina Kraus^57^ and presented at 80 dBA (rms level) through a loudspeaker that was 1 m away from the participant. The stimulus consists of an onset burst (5-msec), a formant transition period (45-msec), and a steady-state period (120-msec). The /da/ stimulus was chosen for several reasons. First, it is widely used in many studies as it is a common syllable in most European languages and produces clear and replicable ABRs^24^. Second, the acoustic characteristics of the stimulus is similar to the click ABR and FFR—it contains a transient and a sustained segments^24^. The sampling rate was 20,000 Hz and filtering range was from 70 to 2,000 Hz (12 dB/octave). A total of six thousand sweeps (three thousand sweeps for each polarity) were obtained for repeatability of the waveforms. Both groups were tested in 0 and +5 dB SNR conditions. To minimize artifacts, participants sat in a comfortable sofa and watched a movie without audio during the testing and artifact rejection criterion was >20 μV. Responses were obtained with negative and positive polarities and later added together to get rid of cochlear microphonic and stimulus artifacts. The total testing time was two hours for the NH listeners and four hours for the HA group. cABR data analysis was completed using the Brainstem Toolbox which is an open source MATLAB-based toolbox developed by Erika Skoe and Nina Kraus at Northwestern University^24^. Frequency domain analysis for the sustained regions (60–170 ms) was performed using Fourier analysis. For all cABR responses, the mean amplitude at the fundamental frequency (F0, 100 ± 5 Hz) was analyzed. F1 (400-720 Hz) was not analyzed as the responses were in the noise floor.

## Data Availability

All relevant data are within the paper and are available upon request.
